# Trends and associated maternal characteristics of antidiabetic medication use among pregnant women in South Korea

**DOI:** 10.1038/s41598-021-83808-7

**Published:** 2021-02-18

**Authors:** Yunha Noh, Seung-Ah Choe, Ju-Young Shin

**Affiliations:** 1 School of Pharmacy, Sungkyunkwan University, 2066 Seobu-ro, Jangan-gu, Suwon, Gyeonggi-do South Korea; 2 Department of Preventive Medicine, Korea University College of Medicine, Seoul, South Korea; 3 Department of Clinical Research Design and Evaluation, SAIHST, Sungkyunkwan University, Seoul, South Korea

**Keywords:** Epidemiology, Therapeutics

## Abstract

The prevalence of diabetes during pregnancy and the need for the treatment are increasing. We aimed to investigate antidiabetic medications (ADM) use among pregnant women and their characteristics. Using Korea’s nationwide healthcare database, we included women aged 15–49 years with births during 2004–2013. The prevalence and secular trend of ADM use were assessed in 3 periods: pre-conception period, first trimester, and second/third trimesters. To compare maternal characteristics between pregnancies with and without ADM prescription, we used the χ^2^ or Fisher’s exact test and Cochran-Armitage trend test. The prescription patterns analyzed by calendar year, age, insurance type, income, area, and medical institution. Of 81,559 pregnancies, 222 (0.27%) and 305 (0.37%) were exposed ADM during pre-conception and pregnancy periods, respectively. ADM prescriptions increased significantly by an 11.3-fold in second/third trimesters, while a 2.9-fold in first trimester. ADM use is more prevalent in women aged older and living in urban areas. Metformin was most used in the pre-conception period, while insulins were most during pregnancy. About 0.4% of women received ADM during pregnancy; a rate was lower than that in western countries. Non-recommended medications were more common in first trimester, which warrants pregnancy screening for women taking ADM.

## Introduction

Medical complications and the use of medications for them during pregnancy should be monitored carefully, as they may have significant impact not only on maternal but also fetal health^1,2^. Although medication use during pregnancy has grown progressively over past decades^3^, pregnant women remain therapeutic orphans, as they are generally excluded from clinical trials due to ethical concerns and potential fetal risk^4,5^.

Pregestational diabetes mellitus (DM) (both types 1 and 2) in pregnancy results in increased risks of pregnancy outcomes such as miscarriage, congenital malformation, and perinatal mortality^6^. Gestational diabetes mellitus (GDM), one of the most common pregnancy complications, is also associated with increased risks of maternal and perinatal complications^7,8^. To reduce the risk of those adverse complications, management of hyperglycemia during pregnancy is essential. All pregnant women in South Korea are tested for DM at their first prenatal visit, and pregnant women who were not previously diagnosed with DM are screened for GDM at 24–28 weeks of gestation by one of the following two criteria: 1) the International Association of the Diabetes and Pregnancy Study Groups (IADPSG) criteria^9^, and 2) Carpenter-Coustan or National Diabetes Data Group (NDDG) criteria^10^.

Management of hyperglycemia during pregnancy begins with nonpharmacologic strategies; however, pharmacological treatment is required when target glucose levels (fasting blood glucose < 95 mg/dL and postprandial blood glucose < 140 mg/dL at 1 h or < 120 mg/dL at 2 h) cannot be achieved through dietary modification and exercise^11^. Although there is little international consensus on management strategies for DM during pregnancy, insulin, metformin, and glyburide are considered pharmacological therapeutic options. Insulin is recommended as the first-line treatment and metformin (and rarely glyburide) may be an alternative if insulin is unable to be used, according to several associations, including the American Diabetes Association (ADA)^12^, the American College of Obstetricians and Gynecologists (ACOG)^13^, the Canadian Diabetes Association (CDA)^14^, and the Korean Diabetes Association (KDA)^11^. The UK’s regulatory agency, the National Institute for Clinical Excellence (NICE), recommended the use of insulin or metformin (as an adjunct or alternative to insulin) before and during pregnancy and advised that metformin is preferred for the treatment of GDM^15^. The New Zealand’s regulatory agency recommended metformin for GDM treatment^16^.

Metformin was associated with a reduced risk of neonatal hypoglycemia and less maternal weight gain than insulin^17,18^. A recent randomized clinical trial showed that metformin offered maternal glycemic and neonatal adiposity benefits when metformin added to a standard regimen of insulin^19^. On the other hand, in a recent meta-analysis, metformin resulted in lower birth weight in neonates but higher BMI in childhood than insulin^20^. Glyburide was associated with a higher rate of macrosomia and neonatal hypoglycemia than insulin or metformin^18^. Despite the recommendations of metformin and glyburide for the treatment of GDM, both were known to cross the placenta and their long-term safety is still unknown. Other oral agents lack safety data on pregnancy. Given the uncertain safety evidence, South Korea’s health regulatory agency suggests all types of oral antidiabetic agents should be used with caution^21^.

Although the prevalence of DM during pregnancy and the need for treatment for them are steadily increasing^22–24^, research on antidiabetic utilization in pregnancy and maternal characteristics receiving them is limited. Therefore, this descriptive drug utilization study was designed to investigate the utilization of antidiabetic medications in women during their pregnancy and pre-conceptional periods and their maternal characteristics related to antidiabetic medication use, in South Korea.

## Materials and methods

### Data source

This descriptive study was conducted using data from the National Health Insurance Service-National Sample Cohort (NHIS-NSC) database, comprising approximately one million people randomly selected from among the entire Korean population of ≥ 50 million between January 1, 2002, and December 31, 2013^25^. All patients in the data are continuously enrolled in the insurance system unless they are disqualified due to emigration or death. Comprehensive data on patients’ sociodemographic factors, diagnoses, medical procedures, and drug prescription records are available in this database. Diagnosis codes were classified according to the *International Classification of Disease 10th revision* (ICD-10). This study was approved by the Institutional Review Board of Sungkyunkwan University (SKKU 2018–03-014). As this study are based on claim data and do not contact with patients, no informed consent was required from the board.

### Study population

The study population was defined as pregnancies in women aged 15–49 years with one or more live births between January 1, 2004, and December 31, 2013, and we reviewed the claim records between January 1, 2002 and December 31, 2013. The history of live births were identified from a domestic procedure code of delivery (R3131-R3148, R4351-R4362, R4380, R4507-R4520, R5001-R5002, RA311-RA318, RA361-RA362, and RA380-RA434); the delivery date was defined as the date that a procedure code of delivery was recorded in the database. As neither the last menstrual period (LMP) nor gestational length were provided in our data, gestational age was estimated based on the delivery date, assigning all pregnancies a fixed duration of 273 days^26^. If a pregnant woman had more than two delivery procedure codes within 273 days, these were considered duplicate records of the same delivery and only the first recorded delivery was considered valid. We identified a total of 81,559 pregnancies among 58,486 women. From the total pregnancies, we identified pregnancies in women who prescribed any antidiabetic medication during pre-conception or pregnancy period, respectively. All pregnancies with live births were retained in the final cohort instead of pregnant women, as maternal characteristics and trend of medication use may be time-dependent.

### Exposure assessment

The drugs of interest were all antidiabetic medications (Anatomical Therapeutic Chemical [ATC] classification code A10), including insulins (human insulin and insulin analogues) and all oral antidiabetic agents (biguanides [metformin only], sulfonylureas, thiazolidinediones, α-glucosidase inhibitors, dipeptidyl peptidase [DPP]-4 inhibitors, meglitinides, glucagon-like peptide [GLP]-1 analogues, and Sodium/glucose cotransporter [SGLT]-2 inhibitors) (Table S1). Combination products were also included in this study, with each drug’s active ingredient considered individually. Among the aforementioned antidiabetic medications, our analysis focused on the three most commonly utilized drug classes (insulins, metformin, and sulfonylureas) and the remaining drug classes were categorized as other oral agents.

Exposure assessment was conducted in following periods: pre-conception period (the year before pregnancy; > 273 days), first trimester (273–184 days), and second or third trimesters (183–8 days before the delivery date) (Figure S1). Medications prescribed within seven days before the delivery date were excluded from analysis as their prescription patterns were likely to have changed for delivery purposes. Study subjects were considered exposed to the drugs of interest if they received at least one prescription of an antidiabetic medication(s) during each of the above-mentioned periods. All medication prescription records from outpatient settings were identified from the NHIS-NSC database.

### Identification of maternal characteristics

The baseline characteristics of the pregnancies were determined and stratified by whether the women were prescribed antidiabetic medications during pregnancy. Maternal age at delivery, insurance type (health insurance and medical aid), income level (low-income [0–3 deciles], middle-income [4–7 deciles] and high-income [8–10 deciles] based on income level deciles) and area (urban and rural) were extracted from the database. Nulliparity and multifetal gestation was determined by the procedure codes of delivery. The presence of chronic hypertension (HTN) (ICD-10 codes: I10-I15 and O10-O11), gestational HTN (O13), preeclampsia-eclampsia (O14-O15), pregestational DM (E10-E14 and O240-O243), GDM (O244), polycystic ovary syndrome (PCOS) (E282), and female infertility (including amenorrhea or irregular menstruation) (N91, N97) was identified by the records of appropriate the ICD-10 code diagnosed during pre-conception and pregnancy periods. The number of physician visits for any reason during pre-conception period was determined as a proxy for health care utilization. To understand the potential underlying causes for each class of antidiabetic prescriptions, we confirmed diagnosis codes for DM, GDM, and PCOS or female infertility using ICD-10 codes, which are corresponding to each antidiabetic prescription.

### Statistical analysis

We calculated absolute standardized differences (aSD) to estimate the size of the difference in baseline characteristics between pregnancies with and without antidiabetic prescriptions during pregnancy^27^. We defined a significant difference between the two groups as aSD greater than 0.1. Descriptive statistics were presented as the numbers and prevalence of pregnancies among women prescribed antidiabetic medications in per-conception period, first trimester, and second or third trimester, separately. The prevalence was calculated as the number of pregnancies in women prescribed any antidiabetic medication, where the denominator was the total number of pregnancies with live births. The prevalence of antidiabetic medication use for each drug class was stratified by calendar year, maternal age at delivery, insurance type, income level, area and medical institution type. The medical institution types were classified according to the number of beds: primary (0–29 beds), secondary (30–99 beds), and tertiary (≥ 100 beds). To compare the maternal characteristics between pregnancies with and without prescriptions of each antidiabetic medication class, the χ2 test or Fisher’s exact test was used for variables of insurance type and area, and the Cochran-Armitage trend test was used for variables of the calendar year, maternal age, and income level. *p* values of < 0.05 were significant. We investigated secular trends in annual drug utilization in the pre-conception period, first trimester, and second or third trimesters. The data analysis of this study was performed using SAS® software, version 9.4 (© 2002–2012 by SAS Institute Inc., Cary, NC, USA).

## Results

**Figure 1:**
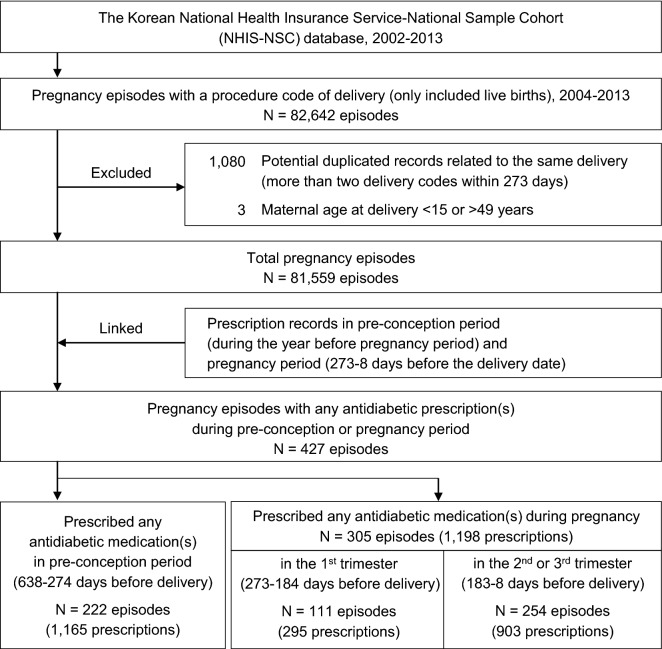
Flow chart for identification of study population.

Among the 81,559 pregnancies, 222 (0.27%) and 305 (0.37%) pregnancies were prescribed any antidiabetic medication(s) during the pre-conception and pregnancy periods, respectively (Fig. 1). A higher proportion of pregnancies in women with antidiabetic prescriptions were to those in the 35–39 years and 40–44 years age group, compared to those without antidiabetic prescribing (30.5% vs. 15.5% at 35–39 years and 6.6% vs. 2.0% at 40–44 years) (Table 1). Moreover, a higher proportion was showed in medical aid beneficiaries (1.6% of antidiabetic exposed vs. 0.3% of their counterparts; aSD = 0.139), those living in urban areas (77.7% vs.70.4%; aSD = 0.168), multifetal gestation (4.6% vs.1.4%; aSD = 0.192). Pregnancies with antidiabetic prescriptions also had more comorbid conditions and physician visits in pre-conception period (12 or more) (46.6% vs 24.9%; aSD = 0.469) compared to their counterparts.

**Table 1:** Comparison of characteristics between pregnancies with and without antidiabetic prescriptions during pregnancy, 2004–2013 (total pregnancies = 81,559).

	Pregnancies	Pregnancies	aSD*
	w/antidiabetic Rx	w/o antidiabetic Rx	
	N (%)	N (%)	
**Pregnancies**	305	81,254	
**Maternal age at delivery (years)**			0.562
15–19	0 (0.0)	260 (0.3)	0.080
20–24	5 (1.6)	3498 (4.3)	0.157
25–29	43 (14.1)	24,336 (30.0)	0.390
30–34	144 (47.2)	38,911 (47.9)	0.014
35–39	93 (30.5)	12,559 (15.5)	0.363
40–44	20 (6.6)	1628 (2.0)	0.226
45–49	0 (0.0)	62 (0.1)	
**Insurance type**			0.131
Health insurance	300 (98.4)	80,974 (99.7)	
Medical aid	5 (1.6)	280 (0.3)	
**Income level**			0.075
Low-income	40 (13.1)	12,547 (15.4)	0.067
Middle-income	155 (50.8)	41,420 (51.0)	0.003
High-income	110 (36.1)	27,287 (33.6)	0.052
**Area**			0.168
Urban	237 (77.7)	57,163 (70.4)	
Rural	68 (22.3)	24,091 (29.6)	
**Nulliparity**	137 (44.9)	41,055 (50.5)	0.112
**Multifetal gestation**	14 (4.6)	1098 (1.4)	0.192
**Comorbid conditions**			
Chronic hypertension	32 (10.5)	958 (1.2)	0.405
Gestational hypertension	11 (3.6)	469 (0.6)	0.213
Preeclampsia-eclampsia	10 (3.3)	807 (1.0)	0.159
Pregestational diabetes mellitus	200 (65.6)	1419 (1.7)	1.831
Gestational diabetes mellitus	58 (19.0)	2260 (2.8)	0.540
PCOS	14 (4.6)	755 (0.9)	0.225
Female infertility	116 (38.0)	19,044 (23.4)	0.320
**Number of physician visits for any reason during pre-conception period**			0.469
0–3	57 (18.7)	23,468 (28.9)	0.241
4–11	106 (34.8)	37,593 (46.3)	0.236
12 or more	142 (46.6)	20,193 (24.9)	0.465
**Exposed to antidiabetic medications during pre-conception period**	100 (32.8)	122 (0.2)	0.980

aSD, absolute standardized difference; Rx, medical prescriptions; PCOS, polycystic ovary syndrome.

*Absolute standardized difference, whereby value > 0.10 indicates significant difference between antidiabetic medication users and non-users during pregnancy.

**Figure 2:**
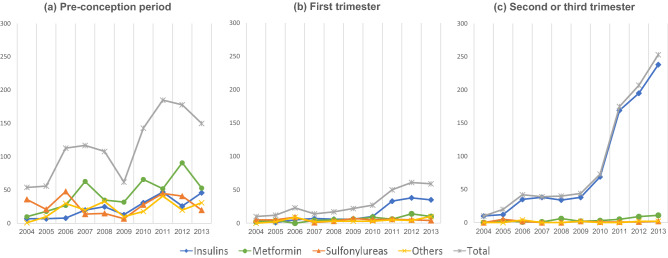
Secular patterns of antidiabetic prescriptions in the pre-conception period, first trimester, and second or third trimesters. *The y axis represents the number of prescriptions for each drug class.

The secular patterns of antidiabetic prescriptions among pregnancies from 2004 to 2013 revealed an overall increasing trend in all periods, especially in the second or third trimesters (Fig. 2). A sharp increase was observed in the second or third trimesters, with insulins most commonly used, indicating that the prescriptions of insulins almost coincided with those of total antidiabetic medications.

Of 222 pregnancies who were prescribed any antidiabetic medication(s) in the pre-conception period, metformin (0.17%) was most commonly used, followed by other oral agents (0.08%), and sulfonylureas (0.07%) (Table 2). The prescription of any antidiabetics increased with maternal age (*p* < 0.001). Insulins were prescribed at a higher proportion in tertiary care (35.3%) than in primary (14.2%) or secondary (13.7%) care. Among women who used antidiabetic medications during the pre-conception period, 56.8% had DM only, 11.3% had DM with other condition(s) of interest, and 29.3% had PCOS or infertility (Table S2).

**Table 2:** Utilization of antidiabetic medications in the pre-conception period by drug class* and maternal characteristics, 2004–2013 (N = 81,559).

	Pregnancy episodes	Any antidiabetic medication use	*p*-value^‡^	Insulins	*p*-value	Metformin	*p*-value	Sulfonylureas	*p*-value	Other oral agents	*p*-value
	N	N (%)^†^		N (%)		N (%)		N (%)		N (%)	
**Total pregnancy episodes**	81,559	222 (0.27)		45 (0.06)		142 (0.17)		58 (0.07)		62 (0.08)	
**Year of delivery**											
2004	6670	6 (0.09)	0.013	1 (0.01)	< 0.001	4 (0.06)	0.005	5 (0.07)	0.873	1 (0.01)	0.554
2005	8177	19 (0.23)		1 (0.01)		11 (0.13)		9 (0.11)		4 (0.05)	
2006	8219	15 (0.18)		3 (0.04)		9 (0.11)		8 (0.10)		5 (0.06)	
2007	9018	28 (0.31)		4 (0.04)		18 (0.20)		2 (0.02)		8 (0.09)	
2008	8278	33 (0.40)		4 (0.05)		16 (0.19)		5 (0.06)		16 (0.19)	
2009	7689	21 (0.27)		3 (0.04)		12 (0.16)		3 (0.04)		7 (0.09)	
2010	7985	22 (0.28)		4 (0.05)		18 (0.23)		5 (0.06)		3 (0.04)	
2011	8651	25 (0.29)		9 (0.10)		14 (0.16)		7 (0.08)		7 (0.08)	
2012	8876	30 (0.34)		8 (0.09)		25 (0.28)		8 (0.09)		5 (0.06)	
2013	7996	23 (0.29)		8 (0.10)		15 (0.19)		6 (0.08)		6 (0.08)	
**Maternal age at delivery (years)**											
20–24	3503	7 (0.20)	< 0.001	2 (0.06)	0.054	5 (0.14)	0.017	1 (0.03)	< 0.001	0	< 0.001
25–29	24,379	45 (0.18)		7 (0.03)		30 (0.12)		10 (0.04)		9 (0.04)	
30–34	39,055	119 (0.30)		23 (0.06)		78 (0.20)		28 (0.07)		37 (0.09)	
35–39	12,652	43 (0.34)		13 (0.10)		22 (0.17)		14 (0.11)		12 (0.09)	
40–44	1648	8 (0.49)		0		7 (0.42)		5 (0.30)		4 (0.24)	
**Insurance type**											
Health insurance	81,274	219 (0.27)	0.043	45 (0.06)	1.000	140 (0.17)	0.089	55 (0.07)	0.001	61 (0.08)	0.195
Medical aid	285	3 (1.05)		0		2 (0.70)		3 (1.05)		1 (0.35)	
**Income level**											
Low-income	12,587	29 (0.23)	0.506	6 (0.05)	0.687	19 (0.15)	0.729	8 (0.06)	0.623	7 (0.06)	0.961
Middle-income	41,575	117 (0.28)		23 (0.06)		81 (0.19)		34 (0.08)		37 (0.09)	
High-income	27,397	76 (0.28)		16 (0.06)		42 (0.15)		16 (0.06)		18 (0.07)	
**Area**											
Urban	57,400	167 (0.29)	0.113	35 (0.06)	0.277	110 (0.19)	0.064	42 (0.07)	0.734	48 (0.08)	0.224
Rural	24,159	55 (0.23)		10 (0.04)		32 (0.13)		16 (0.07)		14 (0.06)	
**Medical institution type**^§^											
Primary care		732		104 (14.2)		274 (37.4)		221 (30.2)		133 (18.2)	
Secondary care		124		17 (13.7)		57 (46.0)		10 (8.1)		40 (32.3)	
Tertiary care		309		109 (35.3)		116 (37.5)		43 (13.9)		41 (13.3)	

*When a pregnant woman was prescribed two or more drug's active ingredient, each ingredient was separated individually.

^†^All percentages are row percentage with pregnancies corresponding to each row.

^‡^The *p*-value s denote comparison between pregnancies with and without prescriptions of each drug class. The χ^2^ test or Fisher’s exact test was used for variables of insurance type and area, and the Cochran-Armitage trend test was used for variables of the calendar year, maternal age, and income level. *p* values of < 0.05 were significant.

^§^In the case of medical institution type, we used the number of prescriptions, not the number of pregnancies.

Any antidiabetic medication(s) was prescribed to women in the first trimester for 111 pregnancies (0.14%) and in the second or third trimesters for 254 pregnancies (0.31%) (Tables 3 and 4). The most commonly used medication in the first trimester was insulins (0.06%), followed by metformin (0.05%) and sulfonylureas (0.03%), while that in the second or third trimesters was insulins (0.29%). In the first trimester, antidiabetic prescriptions increased from 0.07% in 2004 to 0.20% in 2013, a 2.9-fold increase (*p* = 0.001), and from 0.06 to 0.68% in the second or third trimesters, an 11.3-fold increase (*p* < 0.001).

**Table 3:** Utilization of antidiabetic medications during the first trimester, by drug class^*^ and maternal characteristics, 2004–2013 (N = 81,559).

	Pregnancy episodes	Any antidiabetic medication use	*p*-value ^‡^	Insulins	*p*-value	Metformin	*p*-value	Sulfonylureas	*p*-value	Other oral agents	*p*-value
	N	N (%)^†^		N (%)		N (%)		N (%)		N (%)	
**Total pregnancies**	81,559	111 (0.14)		50 (0.06)		43 (0.05)		28 (0.03)		27 (0.03)	
**Year of delivery**											
2004	6670	5 (0.07)	0.001	2 (0.03)	< 0.001	2 (0.03)	0.003	2 (0.03)	0.520	0	0.077
2005	8177	7 (0.09)		1 (0.01)		3 (0.04)		4 (0.05)		2 (0.02)	
2006	8219	9 (0.11)		4 (0.05)		0		5 (0.06)		4 (0.05)	
2007	9018	9 (0.10)		3 (0.03)		3 (0.03)		1 (0.01)		2 (0.02)	
2008	8278	11 (0.13)		4 (0.05)		5 (0.06)		3 (0.04)		2 (0.02)	
2009	7689	11 (0.14)		4 (0.05)		3 (0.04)		2 (0.03)		3 (0.04)	
2010	7985	11 (0.14)		4 (0.05)		7 (0.09)		3 (0.04)		1 (0.01)	
2011	8651	12 (0.14)		6 (0.07)		4 (0.05)		4 (0.05)		4 (0.05)	
2012	8876	20 (0.23)		12 (0.14)		9 (0.10)		2 (0.02)		4 (0.05)	
2013	7996	16 (0.20)		10 (0.13)		7 (0.09)		2 (0.03)		5 (0.06)	
**Maternal age at delivery (years)**											
20–24	3503	3 (0.09)	< 0.001	2 (0.06)	0.012	1 (0.03)	0.009	1 (0.03)	< 0.001	1 (0.03)	0.009
25–29	24,379	17 (0.07)		8 (0.03)		6 (0.02)		3 (0.01)		3 (0.01)	
30–34	39,055	54 (0.14)		23 (0.06)		24 (0.06)		13 (0.03)		14 (0.04)	
35–39	12,652	32 (0.25)		17 (0.13)		10 (0.08)		7 (0.06)		7 (0.06)	
40–44	1648	5 (0.30)		0		2 (0.12)		4 (0.24)		2 (0.12)	
**Insurance type**											
Health insurance	81,274	109 (0.13)	0.058	50 (0.06)	1.000	42 (0.05)	0.140	27 (0.03)	0.093	26 (0.03)	0.090
Medical aid	285	2 (0.70)		0		1 (0.35)		1 (0.35)		1 (0.35)	
**Income level**											
Low-income	12,587	12 (0.10)	0.214	3 (0.02)	0.062	5 (0.04)	0.855	6 (0.05)	0.389	4 (0.03)	0.978
Middle-income	41,575	58 (0.14)		26 (0.06)		26 (0.06)		14 (0.03)		14 (0.03)	
High-income	27,397	41 (0.15)		21 (0.08)		12 (0.04)		8 (0.03)		9 (0.03)	
**Area**											
Urban	57,400	89 (0.16)	0.024	39 (0.07)	0.238	37 (0.06)	0.024	24 (0.04)	0.075	24 (0.04)	0.035
Rural	24,159	22 (0.09)		11 (0.05)		6 (0.02)		4 (0.02)		3 (0.01)	
**Medical institution type**^**§**^											
Primary care		190		85 (44.7)		35 (18.4)		40 (21.1)		30 (15.8)	
Secondary care		25		9 (36.0)		11 (44.0)		1 (4.0)		4 (16.0)	
Tertiary care		78		50 (64.1)		14 (18.0)		8 (10.3)		6 (7.7)	

*When a pregnant woman was prescribed two or more drug's active ingredient, each ingredient was separated individually.

^†^All percentages are row percentage with pregnancies corresponding to each row.

^‡^The *p*-value s denote comparison between pregnancies with and without prescriptions of each drug class. The χ^2^ test or Fisher’s exact test was used for variables of insurance type and area, and the Cochran-Armitage trend test was used for variables of the calendar year, maternal age, and income level. *p* values of < 0.05 were significant.

^§^In the case of medical institution type, we used the number of prescriptions and not the number of pregnancies.

**Table 4:** Utilization of antidiabetic medications during the second or third trimester, by drug class^*^ and maternal characteristics, 2004–2013 (N = 81,559).

	Pregnancy episodes	Any antidiabetic medication use	*p*-value ^‡^	Insulins	*p*-value	Metformin	*p*-value	Sulfonylureas	*p*-value	Other oral agents	*p*-value
	N	N (%)^†^		N (%)		N (%)		N (%)		N (%)	
**Total pregnancies**	81,559	254 (0.31)		237 (0.29)		20 (0.02)		9 (0.01)		6 (0.01)	
**Year of delivery**											
2004	6670	4 (0.06)	< 0.001	4 (0.06)	< 0.001	0	0.003	0	0.874	0	0.353
2005	8177	9 (0.11)		7 (0.09)		1 (0.01)		3 (0.04)		0	
2006	8219	16 (0.19)		13 (0.16)		1 (0.01)		1 (0.01)		2 (0.02)	
2007	9018	13 (0.14)		12 (0.13)		1 (0.01)		0		0	
2008	8278	13 (0.16)		12 (0.14)		1 (0.01)		0		0	
2009	7689	13 (0.17)		13 (0.17)		1 (0.01)		1 (0.01)		1 (0.01)	
2010	7985	25 (0.31)		23 (0.29)		3 (0.04)		1 (0.01)		0	
2011	8651	48 (0.55)		45 (0.52)		3 (0.03)		1 (0.01)		0	
2012	8876	59 (0.66)		54 (0.61)		5 (0.06)		1 (0.01)		2 (0.02)	
2013	7996	54 (0.68)		54 (0.68)		4 (0.05)		1 (0.01)		1 (0.01)	
**Maternal age at delivery (years)**											
20–24	3503	5 (0.14)	< 0.001	5 (0.14)	< 0.001	0	< 0.001	0	0.058	0	< 0.001
25–29	24,379	34 (0.14)		32 (0.13)		2 (0.01)		2 (0.01)		0	
30–34	39,055	119 (0.30)		113 (0.29)		9 (0.02)		3 (0.01)		2 (0.01)	
35–39	12,652	79 (0.62)		72 (0.57)		6 (0.05)		3 (0.02)		2 (0.02)	
40–44	1648	17 (1.03)		15 (0.91)		3 (0.18)		1 (0.06)		2 (0.12)	
**Insurance type**											
Health insurance	81,274	251 (0.31)	0.060	234 (0.29)	0.051	20 (0.02)	1.000	9 (0.01)	1.000	6 (0.01)	1.000
Medical aid	285	3 (1.05)		3 (1.05)		0		0		0	
**Income level**											
Low-income	12,587	32 (0.25)	0.167	30 (0.24)	0.250	3 (0.02)	0.835	0	0.501	0	0.249
Middle-income	41,575	129 (0.31)		122 (0.29)		11 (0.03)		6 (0.01)		3 (0.01)	
High-income	27,397	93 (0.34)		85 (0.31)		6 (0.02)		3 (0.01)		3 (0.01)	
**Area**											
Urban	57,400	198 (0.34)	0.008	185 (0.32)	0.010	16 (0.03)	0.346	8 (0.01)	0.224	5 (0.01)	0.487
Rural	24,159	56 (0.23)		52 (0.22)		4 (0.02)		1 (0.00)		1 (0.00)	
**Medical institution type**^**§**^											
Primary care		339		318 (93.8)		8 (2.4)		8 (2.4)		5 (1.5)	
Secondary care		152		132 (86.8)		17 (11.2)		1 (0.7)		2 (1.3)	
Tertiary care		411		388 (94.4)		16 (3.9)		4 (1.0)		3 (0.7)	

*When a pregnant woman was prescribed two or more drug's active ingredient, each ingredient was separated individually.

^†^All percentages are row percentage with pregnancies corresponding to each row.

^‡^The *p*-values denote comparison between pregnancies with and without prescriptions of each drug class. The χ^2^ test or Fisher’s exact test was used for variables of insurance type and area, and the Cochran-Armitage trend test was used for variables of the calendar year, maternal age, and income level. *p* values of < 0.05 were significant.

^§^In the case of medical institution type, we used the number of prescriptions and not the number of pregnancies.

For both first and second or third trimesters, antidiabetic prescriptions increased with maternal age (*p* < 0.001). Especially, compared to that in the pre-conception period, antidiabetic medication use in the second or third trimesters was doubled in women aged 35–39 and 40–44 years of age (0.34% to 0.62% and 0.49% to 1.03%, respectively), while there was little change in women aged 20–34 years. In both first and second or third trimesters, medical aid beneficiaries used more antidiabetic medications than health insurance subscribers (0.70% vs. 0.13% and 1.05% vs. 0.31%, respectively), but it was not statistically significant (*p* = 0.058 and *p* = 0.060, respectively). In both first and second or third trimesters, pregnant women living in urban areas used more antidiabetic medications than those living in rural areas (*p* = 0.024, and *p* = 0.008, respectively).

Among women who were prescribed antidiabetic medications in the first trimester, 76.6% had DM only, 17.1% had PCOS or infertility, and 3.6% had DM with other condition(s) of interest. Among women who used antidiabetic medications in the second or third trimesters, 59.4% had DM only and 36.6% had GDM only (Table S2).

## Discussion

Of the 81,559 pregnancies, approximately 0.4% of women received antidiabetic medication(s) during pregnancy between 2004 and 2013. Overall, the antidiabetic medication exposures declined from the pre-conception period (0.27%) to the first trimester (0.14%) but increased in the second or third trimesters (0.31%). The number of women receiving antidiabetic medications during pregnancy, especially in the second or third trimester, increased considerably over the ten-year study period. Antidiabetic prescriptions are more prevalent in women who were older and those living in urban areas, with statistical significance. In the pre-conception period, metformin (0.17%) was most used, while insulins were most used (0.06% and 0.29%, respectively) in the first and second or third trimesters. Pregnancies in women prescribed oral antidiabetic agents other than metformin or sulfonylurea were about 0.03% of the total pregnancies in the first trimester and 0.01% in the second or third trimesters.

The prevalence of antidiabetic prescriptions during pregnancy was lower than that in western countries, although careful interpretation is required as their study period, study population, or data source varies among each study. In the US, 3.24% of pregnancies were exposed to antidiabetic medications in second or third trimesters during 2001–2007^28^. In Europe, 2.0% of pregnant women prescribed antidiabetic mediations in the year before, during, or the year following pregnancy between 2004 and 2010^29^. In a recent study, prevalence of any antidiabetic medication use was 3% from under 2% (Denmark, Norway, and Sweden) to above 5% (Australia and US) between 2006 and 2016^30^.

The utilization of antidiabetic medications rose significantly by an 11.3-fold increase in the second and third trimesters, while the increase was more gradual in the first trimester, resulting in a 2.9-fold increase from 2004 to 2013. The increase in antidiabetic medication use might be explained by changes in maternal characteristics. For example, the maternal age at delivery is increasing in Korea, from an average age of first delivery of 27.6 years in 1993 to 31.3 years in 2010^31^. Maternal age is an important risk factor of GDM^32^, and in our study, the prevalence of antidiabetic medication prescriptions increased with maternal age at delivery, an increase more noticeable in the second or third trimesters, in which GDM treatment is initiated. Moreover, the multifetal birth rate also is increasing in Korea, from 1.13% in 1993 to 2.76% in 2010^31^. Multifetal pregnancies were considered a risk factor of GDM^33,34^. We observed that antidiabetic prescriptions in the second or third trimesters increased rapidly in 2011. In Korea, GDM screening has been performed in a two-step approach using Carpenter-Coustan or NDDG criteria; however, in 2011, the KDA additionally adopted a one-step approach using the IADPSG criteria, which might result in an increasing prevalence of GDM^11,35^.

Overall, the antidiabetic medication exposures declined from the pre-conception period (0.27%) to the first trimester (0.14%) but increased in the second or third trimesters (0.31%). The decreased medication use in the first trimester may have been because the first trimester is a crucial period for placental development and organogenesis. As all pregnant women are recommended to undergo GDM screening during the second trimester, it is natural that GDM treatment increased accordingly in the second or third trimesters^36^. In the pre-conception period, metformin use was most prevalent (0.17%). The prevalent use of metformin before pregnancy might be explained by its clinical use. Metformin is the preferred initial pharmacologic agent for type 2 DM and is also used for clinical management of PCOS or infertility as it can induce ovulation^37,38^. A study from the US showed that only 13% of women prescribed metformin in the 4 months before pregnancy were diagnosed with DM and 67% were diagnosed with PCOS or infertility^28^. This finding is different from our results that those diagnosed with DM (42.8%) were more than those diagnosed with PCOS or infertility (30.7%) among women receiving metformin before pregnancy.

In our study, we could not find a significant relationship between socioeconomic deprivation and antidiabetic medication use during pregnancy due to small sample size of medical aid beneficiaries. Previous studies have shown that the association between socioeconomic status and odds of antidiabetic prescriptions or risk of GDM varies for each country. In a large cohort study in Ontario, Canada, the risk of GDM increased among participants with a lower income^39^. Similarly, in a large Scottish cohort, there was an inverse relationship between socioeconomic status and the risk of GDM^40^. However, in a cohort study from the UK, no association was observed between socioeconomic deprivation and the rate of GDM^41^. Chinese^42^ and Saudi Arabian^43^ studies also showed no association between income and GDM. Meanwhile, antidiabetic medication use was more prevalent in pregnant women living in urban areas than that in those living in rural areas, which is similar with the results of previous study^39^. Women living in urban areas had a higher chance of receiving perinatal examinations and were, thus, GDM was more likely to be detected.

Regarding the use of antidiabetic medications before and during pregnancy, the prescription rate and the most prevalent antidiabetic medication in Korea differ from those in the US^28^. The prescription rate in the US was much higher than that in Korea (1.21% vs. 0.27% in the pre-conception period, 1.35% vs. 0.14% in the first trimester, and 3.24% vs. 0.31% in second or third trimesters). In the pre-conception period, the medications used most in the US were metformin (0.84%) and insulins (0.33%), while those in Korea were metformin (0.17%) and other oral agents (0.08%). In the second or third trimesters, more than 90% of Korean women prescribed antidiabetic medications were administered insulins, while insulins (2.45%) and sulfonylureas (0.83%) were most commonly administered in the US.

Metformin was most commonly used in the pre-conception period (0.17%), while insulins were the most prevalent in the first and second or third trimesters (0.06% and 0.29%, respectively). As insulin is the preferred medication for the treatment of hyperglycemia during pregnancy, the utilization of all oral antidiabetic agents decreased as pregnancy progressed, in line with an increase in the prescription of insulin. Nevertheless, oral antidiabetic agents were commonly used and oral agents other than metformin, whose safety have not been proven, were used during pregnancy. Of note, the use of glyburide in the first trimester may be a public health concern, as several studies showed that glyburide increase the risks of neonatal intensive care unit (NICU) admissions, macrosomia, large for gestational age and pre-eclampsia in the mother^44^. Prescriptions of these non-recommended agents may have occurred either when healthcare providers or pregnant women were unaware of their pregnancy status. Hence, it is both necessary to confirm the evidence of drug safety and to improve the system, including alerts for the prescription or dispensing of drugs not recommended to pregnant women or checking pregnancy status beforehand.

This study has several strengths. Firstly, we described the drug utilization using a nationally representative NHIS-NSC database based on systematic stratified random sampling. Secondly, this is the study to explore antidiabetic medication use among pregnant women, which included all kinds of antidiabetic medications in Korea. However, this study also had some limitations. First, the medication history from claim data should not be interpreted as the actual use due to the potential for patient non-compliance. Second, drug exposures in study periods can be misclassified due to a lack of data on gestational age. Hence, we used a previously validated algorithm to estimate the gestational age at birth^26,45^. Third, we included only pregnancies with live births, which can result in an under-estimation of the prevalence of antidiabetic prescriptions by excluding the cases with drug-induced stillbirths or abortions. Fourth, as the data used in this study was only available as of 2013, we could not assess the latest pattern of antidiabetic medication use during pregnancy but we investigated the 10-year longitudinal trends of antidiabetic prescriptions among pregnant women.

In summary, about 0.4% of women in South Korea received antidiabetic medications during pregnancy between 2004 and 2013; a rate was substantially lower than those in western countries. Antidiabetic medications use during pregnancy rose dramatically, especially in the second and third trimesters. Oral antidiabetic agents which were not recommended to pregnant women were more commonly prescribed in the first trimester than in the second or third trimesters. Considering the possible teratogenicity of the medications during early pregnancy, this finding warrants the necessity of pregnancy screening for women of childbearing age who are taking antidiabetic medications. Since the safety information of many antidiabetic medications is unclear, these medications should be used carefully in this population until evidence of their safety is established.

## Ethical approval

This study was approved by the Institutional Review Board of Sungkyunkwan University (SKKU 2018-03-014). As this study are based on claim data and do not contact with patients, no informed consent was required from the board. All research was conducted in accordance with guidelines and regulations of the institutional and national research committee and with the 1964 Helsinki declaration and its later amendments or comparable ethical standards.

## Supplementary Information

Supplementary Information.

## Data Availability

Our study used the National Health Insurance Service-National Sample Cohort (NHIS-NSC), established by the NHIS of South Korea. The NHIS forbids the transfer, rent, or sale of the database to any third party other than the researcher, who obtained the approval for the provided database, due to privacy or ethical policy.

## References

[1] Mitchell AA (2011). Adverse drug reactions in utero: perspectives on teratogens and strategies for the future. Clin. Pharmacol. Ther..

[2] Egan AM, Murphy HR, Dunne FP (2015). The management of type 1 and type 2 diabetes in pregnancy. QJM.

[3] Mitchell AA (2011). Medication use during pregnancy, with particular focus on prescription drugs: 1976–2008. Am. J. Obstet. Gynecol..

[4] Mazer-Amirshahi M, Samiee-Zafarghandy S, Gray G, van den Anker JN (2014). Trends in pregnancy labeling and data quality for US-approved pharmaceuticals. Am. J. Obstet. Gynecol..

[5] Shields KE, Lyerly AD (2013). Exclusion of pregnant women from industry-sponsored clinical trials. Obstet. Gynecol..

[6] Abell SK, Nankervis A, Khan KS, Teede HJ (2016). Type 1 and Type 2 diabetes preconception and in pregnancy: health impacts, influence of obesity and lifestyle, and principles of management. Semin. Reprod. Med..

[7] Metzger BE (2008). Hyperglycemia and adverse pregnancy outcomes. N. Engl. J. Med..

[8] Landon MB (2009). A multicenter, randomized trial of treatment for mild gestational diabetes. N. Engl. J. Med..

[9] Metzger BE (2010). International association of diabetes and pregnancy study groups recommendations on the diagnosis and classification of hyperglycemia in pregnancy. Diabetes Care.

[10] Vandorsten JP (2013). NIH consensus development conference: diagnosing gestational diabetes mellitus. NIH Consens. State Sci. Statements.

[11] Korean Diabetes Association (2019). Treatment guideline for diabetes.

[12] American Diabetes Association. 14. Management of Diabetes in Pregnancy: Standards of Medical Care in Diabetes—2021. *Diabetes Care* **44**, S200-S210 (2021). 10.2337/dc21-S014 33298425

[13] ACOG Practice Bulletin No. 190: Gestational Diabetes Mellitus. *Obstet. Gynecol.* **131**, e49–e64, doi:10.1097/aog.0000000000002501 (2018). 10.1097/AOG.0000000000002501 29370047

[14] Feig DS (2018). Diabetes and pregnancy. Can. J. Diabetes.

[15] National Institute for Health and Care Excellence. Diabetes in pregnancy: management from preconception to the postnatal period. https://www.nice.org.uk/guidance/ng3 (2015). 32212588

[16] Ministry of Health (2014). Diabetes in Pregnancy: Quick Reference Guide for Health Professionals on the Screening, Diagnosis and Treatment of Gestational Diabetes in New Zealand.

[17] Nachum Z (2017). Glyburide versus metformin and their combination for the treatment of gestational diabetes mellitus: a randomized controlled study. Diabetes Care.

[18] Balsells M (2015). Glibenclamide, metformin, and insulin for the treatment of gestational diabetes: a systematic review and meta-analysis. BMJ.

[19] Feig DS (2020). Metformin in women with type 2 diabetes in pregnancy (MiTy): a multicentre, international, randomised, placebo-controlled trial. Lancet Diabetes Endocrinol..

[20] Tarry-Adkins JL, Aiken CE, Ozanne SE (2019). Neonatal, infant, and childhood growth following metformin versus insulin treatment for gestational diabetes: a systematic review and meta-analysis. PLoS Med..

[21] Health Insurance Review and Assessment Service. Drug Utilization Review (DUR) Service (2008).

[22] Ferrara A (2007). Increasing prevalence of gestational diabetes mellitus: a public health perspective. Diabetes Care.

[23] Abouzeid M (2014). A population-based observational study of diabetes during pregnancy in Victoria, Australia, 1999–2008. BMJ Open.

[24] Cho GJ (2015). Secular trends of gestational diabetes mellitus and changes in its risk factors. PLoS ONE.

[25] Lee J, Lee JS, Park SH, Shin SA, Kim K (2017). Cohort profile: The National Health Insurance Service-National Sample Cohort (NHIS-NSC), South Korea. Int. J. Epidemiol..

[26] Margulis AV (2013). Algorithms to estimate the beginning of pregnancy in administrative databases. Pharmacoepidemiol. Drug Saf..

[27] Austin PC (2009). Using the standardized difference to compare the prevalence of a binary variable between two groups in observational research. Commun. Stat. Simul. Comput..

[28] Lawrence JM (2013). Prevalence, trends, and patterns of use of antidiabetic medications among pregnant women, 2001–2007. Obstet. Gynecol..

[29] Charlton RA (2016). Prescribing of antidiabetic medicines before, during and after pregnancy: a study in seven European Regions. PLoS ONE.

[30] Cesta CE (2019). Antidiabetic medication use during pregnancy: an international utilization study. BMJ Open Diabetes Res. Care.

[31] Lim JW (2011). The changing trends in live birth statistics in Korea, 1970 to 2010. Korean J. Pediatr..

[32] Lean SC, Derricott H, Jones RL, Heazell AEP (2017). Advanced maternal age and adverse pregnancy outcomes: a systematic review and meta-analysis. PLoS ONE.

[33] Rauh-Hain JA (2009). Risk for developing gestational diabetes in women with twin pregnancies. J. Matern. Fetal. Neonatal. Med..

[34] Batra S, Sjoberg NO, Aberg A (1978). Human placental lactogen, estradiol-17beta, and progesterone levels in the third trimester and their respective values for detecting twin pregnancy. Am. J. Obstet. Gynecol..

[35] Mayo K, Melamed N, Vandenberghe H, Berger H (2015). The impact of adoption of the international association of diabetes in pregnancy study group criteria for the screening and diagnosis of gestational diabetes. Am. J. Obstet. Gynecol..

[36] Moyer VA (2014). Screening for gestational diabetes mellitus: U.S. Preventive Services Task Force recommendation statement. Ann. Intern. Med..

[37] American Diabetes Association (2017). Standards of medical care in diabetes-2017 abridged for primary care providers. Clin. Diabetes.

[38] Lord JM, Flight IH, Norman RJ (2003). Insulin-sensitising drugs (metformin, troglitazone, rosiglitazone, pioglitazone, D-chiro-inositol) for polycystic ovary syndrome. Cochrane Database Syst. Rev..

[39] Feig DS, Zinman B, Wang X, Hux JE (2008). Risk of development of diabetes mellitus after diagnosis of gestational diabetes. CMAJ.

[40] Collier A (2017). Reported prevalence of gestational diabetes in Scotland: the relationship with obesity, age, socioeconomic status, smoking and macrosomia, and how many are we missing?. J. Diabetes Investig..

[41] Janghorbani M, Stenhouse EA, Jones RB, Millward BA (2006). Is neighbourhood deprivation a risk factor for gestational diabetes mellitus?. Diabetes Med..

[42] Song L (2017). Socio-economic status and risk of gestational diabetes mellitus among Chinese women. Diabetes Med..

[43] Al-Rubeaan K (2014). A community-based survey for different abnormal glucose metabolism among pregnant women in a random household study (SAUDI-DM). BMJ Open.

[44] Malek R, Davis SN (2016). Pharmacokinetics, efficacy and safety of glyburide for treatment of gestational diabetes mellitus. Expert Opin. Drug. Metab. Toxicol..

[45] Eberg M, Platt RW, Filion KB (2017). The estimation of gestational age at birth in database studies. Epidemiology.

