# Supplementary material for: Trends and associated maternal characteristics of antidiabetic medication use among pregnant women in South Korea
Source: Sci Rep. 2021 Feb 18;11:4159. doi: 10.1038/s41598-021-83808-7 (PMC7892865; doi:10.1038/s41598-021-83808-7)
Supplement: Supplementary file 1 — Supplementary Information. [file 41598_2021_83808_MOESM1_ESM.docx]

**Trends and associated maternal characteristics of antidiabetic medication use among pregnant women in South Korea**

Yunha Noh, PharmD^1^; Seung-Ah Choe, MD, PhD^2^; Ju-Young Shin, PhD^1,3^

^1^School of Pharmacy, Sungkyunkwan University, Suwon, South Korea

^2^Department of Preventive Medicine, Korea University College of Medicine, Seoul, South Korea

^3^Department of Clinical Research Design & Evaluation, SAIHST, Sungkyunkwan University, Seoul, South Korea

**Corresponding author**:

Ju-Young Shin, PhD, Associate Professor

School of Pharmacy, Sungkyunkwan University

2066 Seobu-ro, Jangan-gu, Suwon, Gyeonggi-do, South Korea.

Tel: 82-31-290-7702; Fax: 82-31-292-8800

Email: [shin.jy@skku.edu](mailto:shin.jy@skku.edu)

ORCID: 0000-0003-1010-7525

**Short title:** Antidiabetic medication use in pregnancy

**Keywords**: Hypoglycemic agents, drug utilization, epidemiology, diabetes mellitus, pregnancy

**Word count (abstract):** 199

**Word count (main text):** 3,472

**Number of tables**: 4

**Number of figures:** 2

**Supplementary Figure S1.** Scheme for pre-conception, first trimester, and second or third trimester


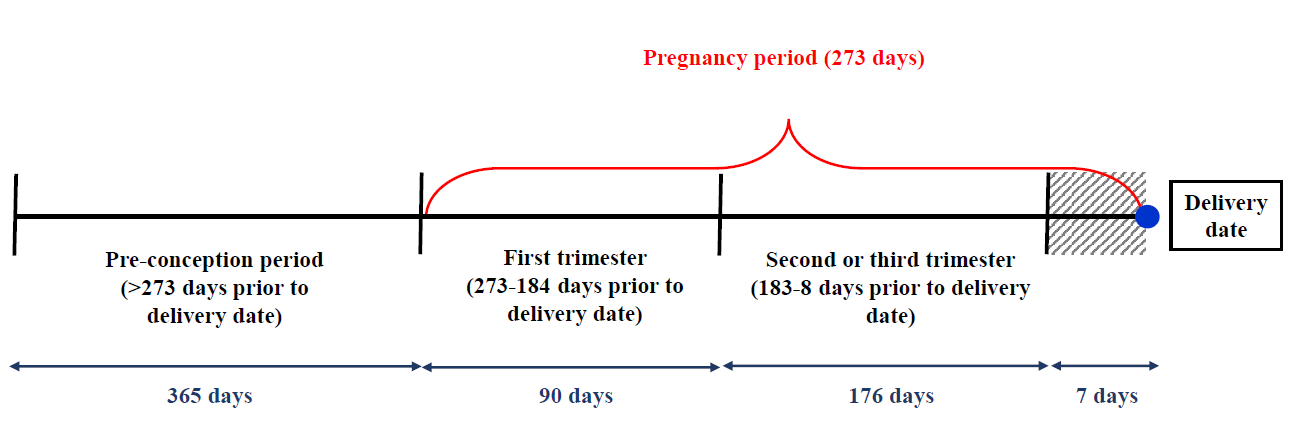


| **Supplementary Table S1.** Utilization of each ingredient in antidiabetic medications^*^ before and during pregnancy and approval dates | | | | | | | | |
| --- | --- | --- | --- | --- | --- | --- | --- | --- |
|  | **Pre-conception** | | **First trimester** | | **Second or third** | | **Approval date^‡^** |  |
|  | **period** | |  |  | **trimester** | |  |  |
|  | **N** | **(%)**^†^ | **N** | **(%)** | **N** | **(%)** |  |  |
| **Any antidiabetic medication** | 222 | (0.27) | 111 | (0.14) | 254 | (0.31) |  |  |
| **Insulins** |  |  |  |  |  |  |  |  |
| Human insulin (intermediate-acting) | 23 | (0.03) | 30 | (0.04) | 113 | (0.14) |  |  |
| Insulin Lispro (rapid-acting) | 19 | (0.02) | 20 | (0.02) | 86 | (0.11) |  |  |
| Insulin Aspart (rapid-acting) | 8 | (0.01) | 7 | (0.01) | 60 | (0.07) | 2002-10-31 |  |
| Insulin Glulisine (rapid-acting) | 2 | (0.00) | 1 | (0.00) | 2 | (0.00) | 2005-03-25 |  |
| Insulin Glargine (long-acting) | 14 | (0.02) | 16 | (0.02) | 23 | (0.03) | 2001-04-04 |  |
| Insulin Detemir (long-acting) | 1 | (0.00) | 3 | (0.00) | 15 | (0.02) | 2006-03-16 |  |
| **Biguanides** |  |  |  |  |  |  |  |  |
| Metformin | 142 | (0.17) | 43 | (0.05) | 20 | (0.02) |  |  |
| **Sulfonylureas** | 0 |  | 0 |  | 0 |  |  |  |
| Glimepiride | 47 | (0.06) | 26 | (0.03) | 7 | (0.01) |  |  |
| Gliclazide | 11 | (0.01) | 2 | (0.00) | 0 |  |  |  |
| Glyburide (Glibenclamide) | 8 | (0.01) | 1 | (0.00) | 1 | (0.00) |  |  |
| Glipizide | 1 | (0.00) | 1 | (0.00) | 1 | (0.00) |  |  |
| **Thiazolidinediones** |  |  |  |  |  |  |  |  |
| Rosiglitazone | 28 | (0.03) | 7 | (0.01) | 1 | (0.00) | 2000-07-01 |  |
| Pioglitazone | 7 | (0.01) | 4 | (0.00) | 1 | (0.00) | 2001-01-16 |  |
| **α-Glucosidase inhibitors** |  |  |  |  |  |  |  |  |
| Voglibose | 9 | (0.01) | 4 | (0.00) | 1 | (0.00) |  |  |
| Acarbose | 7 | (0.01) | 5 | (0.01) | 0 |  |  |  |
| Miglitol | 0 |  | 0 |  | 0 |  | 2001-11-01 |  |
| **DPP-4 inhibitors** |  |  |  |  |  |  |  |  |
| Vildagliptin | 6 | (0.01) | 3 | (0.00) | 1 | (0.00) | 2007-12-28 |  |
| Sitagliptin | 3 | (0.00) | 3 | (0.00) | 2 | (0.00) | 2007-09-21 |  |
| Saxagliptin | 1 | (0.00) | 0 |  | 0 |  | 2011-01-24 |  |
| Linagliptin | 0 |  | 0 |  | 0 |  | 2011-09-14 |  |
| Gemigliptin | 0 |  | 0 |  | 0 |  | 2012-06-27 |  |
| Alogliptin | 0 |  | 0 |  | 0 |  | 2013-05-31 |  |
| **Meglitinides** |  |  |  |  |  |  |  |  |
| Nateglinide | 4 | (0.00) | 2 | (0.00) | 1 | (0.00) | 2000-03-15 |  |
| Repaglinide | 2 | (0.00) | 0 |  | 0 |  |  |  |
| Mitiglinide | 1 | (0.00) | 0 |  | 0 |  | 2006-02-21 |  |
| **GLP-1 analogues** |  |  |  |  |  |  |  |  |
| Exenatide | 0 |  | 0 |  | 0 |  | 2008-05-22 |  |
| **SGLT-2 inhibitors** |  |  |  |  |  |  |  |  |
| Dapagliflozin | 0 |  | 0 |  | 0 |  | 2013-11-26 |  |
| ^*^When a pregnant woman was prescribed two or more drug's active ingredient, each ingredient was separated individually. | | | | | | | | |
| ^†^All percentages were calculated with the total pregnancy episodes in 2004-2013 as the denominator (81,559). | | | | | | | | |
| ^‡^ Approval dates are listed for dates after January 1, 2000. Recently released drugs (e.g., insulin degludec, GLP-1 analogues (lixisenatide, albiglutide, dulaglutide), and SGLT-2 inhibitors (empagliflozin)) were not included in this study. | | | | | | | | |
| **Abbreviation:** DPP, dipeptidyl peptidase. GLP, Glucagon-like peptide. SGLT, Sodium/glucose cotransporter**.** | | | | | | | | |

**Supplementary Table S2.** Diagnosis codes corresponding to antidiabetic prescriptions during each period, 2004-2013 (N=81,559)

|  | **pregnancy episodes** | **DM only** | | **GDM only** | | **DM plus PCOS or infertility** | | **PCOS or infertility** | | **Unknown** | |
| --- | --- | --- | --- | --- | --- | --- | --- | --- | --- | --- | --- |
|  |  | **N** | **(%)^*^** | **N** | **(%)** | **N** | **(%)** | **N** | **(%)** | **N** | **(%)** |
| Pre-conception period | 222 | 126 | (56.8) | - |  | 25 | (11.3) | 65 | (29.3) | 6 | (2.7) |
| First trimester | 111 | 85 | (76.6) | 3 | (2.7) | 4 | (3.6) | 19 | (17.1) | 0 | (0.0) |
| Second or third trimester | 254 | 151 | (59.4) | 93 | (36.6) | 3 | (1.2) | 0 | (0.0) | 7 | (2.8) |
| ^*^All percentages were calculated with the total pregnancy episodes in each period (pre-conception period, first trimester, or second or third trimesters) as the denominator. | | | | | | | | | | | |
| **Abbreviation:** GDM, Gestational diabetes mellitus. DM, Diabetes mellitus. PCOS, Polycystic ovary syndrome. | | | | | | | | | | | |
